# Application of the Single-Molecule Real-Time Technology (SMRT) for Identification of HKαα Thalassemia Allele

**DOI:** 10.1093/labmed/lmac065

**Published:** 2022-09-02

**Authors:** Min Zhang, Zhaodong Lin, Meihuan Chen, Yali Pan, Yanhong Zhang, Lingji Chen, Na Lin, Yuanyuan Ren, Hongjin Jia, Meiying Cai, Liangpu Xu, Hailong Huang

**Affiliations:** Medical Genetic Diagnosis and Therapy Center of Fujian Maternity and Child Health Hospital College of Clinical Medicine for Obstetrics & Gynecology and Pediatrics, Fujian Medical University, Fujian Provincial Key Laboratory of Prenatal Diagnosis and Birth Defect, Fuzhou, China; Department of Clinical Laboratorial Examination, The First Hospital Affiliated to Fujian Medical University, Fuzhou, China; Medical Genetic Diagnosis and Therapy Center of Fujian Maternity and Child Health Hospital College of Clinical Medicine for Obstetrics & Gynecology and Pediatrics, Fujian Medical University, Fujian Provincial Key Laboratory of Prenatal Diagnosis and Birth Defect, Fuzhou, China; Medical Technology and Engineering College of Fujian Medical University, Fuzhou, China; Fujian University of Traditional Chinese Medicine, Fuzhou, China; Medical Genetic Diagnosis and Therapy Center of Fujian Maternity and Child Health Hospital College of Clinical Medicine for Obstetrics & Gynecology and Pediatrics, Fujian Medical University, Fujian Provincial Key Laboratory of Prenatal Diagnosis and Birth Defect, Fuzhou, China; Medical Genetic Diagnosis and Therapy Center of Fujian Maternity and Child Health Hospital College of Clinical Medicine for Obstetrics & Gynecology and Pediatrics, Fujian Medical University, Fujian Provincial Key Laboratory of Prenatal Diagnosis and Birth Defect, Fuzhou, China; Berry Genomics Corporation, Beijing, China; Berry Genomics Corporation, Beijing, China; Medical Genetic Diagnosis and Therapy Center of Fujian Maternity and Child Health Hospital College of Clinical Medicine for Obstetrics & Gynecology and Pediatrics, Fujian Medical University, Fujian Provincial Key Laboratory of Prenatal Diagnosis and Birth Defect, Fuzhou, China; Medical Genetic Diagnosis and Therapy Center of Fujian Maternity and Child Health Hospital College of Clinical Medicine for Obstetrics & Gynecology and Pediatrics, Fujian Medical University, Fujian Provincial Key Laboratory of Prenatal Diagnosis and Birth Defect, Fuzhou, China; Medical Genetic Diagnosis and Therapy Center of Fujian Maternity and Child Health Hospital College of Clinical Medicine for Obstetrics & Gynecology and Pediatrics, Fujian Medical University, Fujian Provincial Key Laboratory of Prenatal Diagnosis and Birth Defect, Fuzhou, China

**Keywords:** single-molecule real-time technology (SMRT), Hong Kongαα, thalassemia, genetic diagnosis, α-globin, gap-PCR

## Abstract

**Objective:**

Single-molecule real-time technology (SMRT) is a sequencing technology using the DNA polymerases and fluorescently tagged nucleotides to accurately sequence DNA strands. The purpose of this study was to evaluate the detection accuracy of SMRT for identification of the Hong Kongαα (HKαα) thalassemia allele.

**Methods:**

We conducted a blinded study of 33 samples of known HKαα alleles. These alleles were detected using SMRT to evaluate accuracy.

**Results:**

We conducted a blinded study of 33 known HKαα samples and found all HKαα variants detected by SMRT to be concordant with those independently assigned by gap-polymerase chain reaction (gap-PCR), reverse dot blot hybridization, and 2-round nested PCR. In addition, SMRT detected 2 β-thalassemia variants that were missed by conventional techniques.

**Conclusion:**

The results demonstrate that SMRT offers a higher detection accuracy of thalassemia rare and new loci. It is an efficient, reliable, and broad-spectrum test that can be widely used for thalassemia screening in the clinic.

Thalassemia is an inherited hematologic disorder characterized by a decrease in or absence of hemoglobin chain synthesis. It is one of the most commonly occurring monogenic disorders, with clinical severity varying from almost asymptomatic to lethal hemolysis.^1–3^ Conventional polymerase chain reaction (PCR) kits currently used in the clinic for the diagnosis of thalassemia mainly include 4 α-globin gene deletions, 3 α-globin point mutations of the α-thalassemia allele, and 17-point mutations of the β-thalassemia allele, which are far from sufficient for the detection of other rare and new variant loci. Such a limited detection scope may lead to inaccurate or missed diagnoses of thalassemia.

The HKαα allele is a rearrangement crossover of the α-globin gene cluster containing both –α ^3.7^ and ααα ^anti4.2^ crossover junctions.^4^ The rate of the HKαα carriers was 0.07%~0.33% and 2.27%~8.81% in –α ^3.7^ positive samples.^5–8^ Previous studies on the hematologic phenotype of HKαα/αα carriers showed that erythrocyte and hemoglobin electrophoresis parameters were not significantly different from those of normal subjects and present a normal phenotype. In addition, the hematologic parameters of --^SEA^/HKαα showed obvious characteristics of α-thalassemia, which are similar to --^SEA^/αα in manifestations without significant hematological differences.^4–10^ The detection area of the conventional thalassemia gene kit does not include ααα ^anti4.2^ variations, and the results of gap-polymerase chain reaction (gap-PCR) indicating a –α ^3.7^/αα condition may actually be –α ^3.7^/αα, –α ^3.7^/HKαα, or HKαα/αα. Similarly, a result of --^SEA^/HKαα may be misdiagnosed as HbH, which may lead to inaccurate genetic counseling and unnecessary invasive prenatal diagnosis. Hence, there is an urgent need for the development and application of newer methods to accurately diagnose rare and novel thalassemia alleles like HKαα.

In recent years, single-molecule real-time technology (SMRT) has been proven to be of potential clinical value in the detection of thalassemia. PacBio SMRT uses a dumbbell library structure to sequence the insert for multiple times and takes the advantage of circular consensus sequencing (CCS) to achieve high-fidelity sequencing for single nucleotide variants (SNVs) and small insertions and deletions (indels). With long-read sequencing, SMRT can detect structural variations and distinguish cis- and trans-configuration for 2 or more variants. Full-length sequencing of HBA1/2 and HBB genes associated with thalassemia using SMRT provided complete variation information of the 2 alleles.^11^ Further, SMRT increased the positive detection rate of the thalassemia gene by 4.9% to 9.91% compared to the conventional genetic techniques.^12,13^ This has helped confirm that SMRT is a scalable, accurate, and cost-effective method for genotyping. Recently, an increasing number of false-negative and false-positive results obtained due to the limitations of traditional diagnostic techniques have been uncovered using SMRT.^12,13^ Given the enhanced accuracy of SMRT compared to conventional techniques, we conducted a blinded study of 33 samples of known HKαα alleles. These alleles were detected using SMRT, and the results illustrated the efficiency, reliability, and broad-spectrum applicability of SMRT in the diagnosis of common and rare variants of thalassemia in the clinical setting.

## Materials and Methods

### Ethical Statement

The study was approved by the Clinical Ethics Committee of the Fujian Provincial Maternity and Children’s Hospital. All procedures were performed in accordance with the Declaration of Helsinki and international and national guidelines for human studies. Written informed consent was obtained from all participants following a detailed description of the purpose of the study.

### Sample Collection

A total of 33 samples of the HKαα allele were collected from February 2016 to December 2019 from the Fujian Provincial Maternity and Children’s Hospital, Fuzhou, China. The study was approved by the Clinical Ethics Committee of the Fujian Provincial Maternity and Children’s Hospital. Following a blind approach, all samples of the HKαα allele were detected by SMRT, and the detection accuracy was determined by comparing it to that obtained post gap-PCR, reverse dot blot hybridization (RDB), and 2-round nested PCR, as described previously.^7^

### Gene Amplification and DNA Libraries

Genomic DNA was amplified by PCR using primers covering the majority of known structural variations, SNVs, and indels in the *HBA1*, *HBA2*, and *HBB* genes. Barcoded adaptors were added to the PCR products using a 1-step end-repair and ligation reaction to construct the prelibraries. Prelibraries were then pooled together with equal mass and converted to the SMRT bell library using Sequel Binding and Internal Ctrl Kit 3.0 (Pacific Biosciences). The CCS mode of the Sequel II platform (Pacific Biosciences) was used to sequence the SMRT bell library. Subreads were processed to CCS reads using CCS software (Pacific Biosciences) and debarcoded using LIMA in the PB Bioconda package (Pacific Biosciences). After alignment of the debarcoded CCS reads to genome build hg38 using PBMN2 (Pacific Biosciences), structural variations were identified according to HbVar, Ithanet, and LOVD databases; and SNVs and indels were identified using FreeBayes1.3.4 (https://www.geneious.com/plugins/freebayes; Biomatters).

## Results

### Sample Identification and Genotyping Data

**FIGURE 1.**
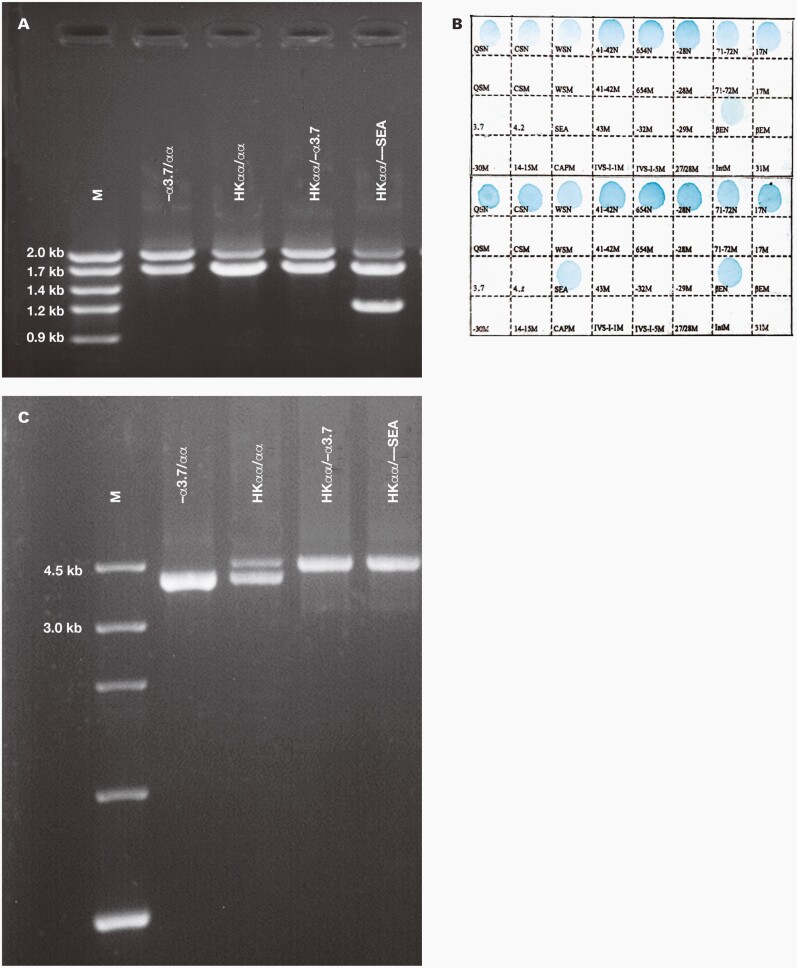
Result of the HKαα allele by gap-polymerase chain reaction (PCR), reverse dot blot hybridization (RDB), and nested PCR. A, Electrophoretogram of the gap-PCR. B, Result of the RDB with –α ^37^/αα and –α ^37^, --^SEA^ and normal α2 alleles. C, Electrophoretogram of the nested PCR.

A total of 33 HKαα allele samples were sequenced and verified using the conventional gap-PCR, RDB, and 2-round nested PCR. Out of the 33 samples, a total of 26 were cases of HKαα/αα compound normal β-thalassemia genotypes, 1 case was HKαα/αα compound HBB:c.316-197C > T Hete, 3 cases were HKαα/αα compound HBB: c.126_129delCTTT Hete, 2 cases were –α ^3.7^/HKαα compound normal β-thalassemia genotypes, 1 case was -- ^SEA^/HKαα compound normal β-thalassemia genotypes, and 1 case was --^SEA^/HKαα compound HBB:c.316-45G > C Hete (**FIGURE 1**). Among the 33 HKαα allele carriers, 9 cases had normal hematological data (**TABLE 1**).

**TABLE 1.** The 33 Samples Carrying the HKαα Allele Detected by Gap-PCR, RDB, and 2-Round Nested PCR

Samples	Sex	Age (y)	RBC (x10^12^/L)	Hb (g/L)	MCV (fL)	MCH (pg)	HbA2 (%)	α Genotype	β Genotype
1^a^	F	26	4.30	125	78.4	29.1	2.7	HKαα/αα	β ^N^/β ^N^
2^a^	F	30	4.05	120	83.5	29.6	2.7	HKαα/αα	β ^N^/β ^N^
3	F	25	4.20	118	76.7	25.6	2.4	HKαα/αα	β ^N^/β ^N^
4	M	28	5.10	140	83.1	28.8	2.6	HKαα/αα	β ^N^/β ^N^
5	M	3	—	—	—	—	2.9	HKαα/αα	β ^N^/β ^N^
6^a^	F	24	3.27	90	84.4	27.5	2.2	HKαα/αα	β ^N^/β ^N^
7^a^	F	31	4.34	104	75.3	24	2.3	HKαα/αα	β ^N^/β ^N^
8	M	28	5.34	159	84.8	29.8	2.6	HKαα/αα	β ^N^/β ^N^
9	F	27	4.36	119	80	27.3	2.4	HKαα/αα	β ^N^/β ^N^
10^a^	F	32	4.57	113	70.9	24.7	2.5	HKαα/αα	β ^N^/β ^N^
11^a^	F	30	3.50	103	88	29.4	2.6	HKαα/αα	β ^N^/β ^N^
12	M	42	5.11	139	82.2	27.9	2.9	HKαα/αα	β ^N^/β ^N^
13^a^	F	22	4.1	108	75.8	25.8	2.5	HKαα/αα	β ^N^/β ^N^
14^a^	F	31	4.15	123	81.2	29.6	2.7	HKαα/αα	β ^N^/β ^N^
15^a^	F	27	4.61	105	74.4	22.8	2.1	HKαα/αα	β ^N^/β ^N^
16^a^	F	34	4.68	131	81.6	28	3	HKαα/αα	β ^N^/β ^N^
17^a^	F	29	4.61	133.1	79.4	28.9	2.9	HKαα/αα	β ^N^/β ^N^
18^a^	F	27	4.28	116	80.4	27.1	2.6	HKαα/αα	β ^N^/β ^N^
19^a^	F	25	3.58	108	89.4	30.2	2.4	HKαα/αα	β ^N^/β ^N^
20^a^	F	26	4.30	120	82	27.3	2.6	HKαα/αα	β ^N^/β ^N^
21	F	28	3.65	114	89.6	31.2	2.6	HKαα/αα	β ^N^/β ^N^
22	M	28	4.79	141	93.1	29.4	2.6	HKαα/αα	β ^N^/β ^N^
23	M	23	5.29	147	92.2	29.1	2.4	HKαα/αα	β ^N^/β ^N^
24	F	47	4.21	93	75.3	22.1	2.5	HKαα/αα	β ^N^/β ^N^
25^a^	F	29	4.95	125	78.8	25.3	2.6	HKαα/αα	β ^N^/β ^N^
26^a^	F	26	5.56	107	59.4	19.2	5.8	HKαα/αα	β ^Codons 41/42^/β ^N^
27	M	3	—	—	—	—	—	HKαα/αα	β ^Codons 41/42^/β ^N^
28	F	28	4.17	95	72	23.9	4.8	HKαα/αα	β ^Codons 41/42^/β ^N^
29^a^	F	43	4.35	90	63.2	20.7	5.1	HKαα/αα	β ^IVS-II-654^/β ^N^
30^a^	F	26	4.70	125	80.4	26.6	3.1	HKαα/–α ^3.7^	β ^N^/β ^N^
31	M	4	5.00	123	80.2	27.3	2.9	HKαα/–α ^3.7^	β ^N^/β ^N^
32	F	27	4.80	119	66.4	30.1	2.4	HKαα/--^SEA^	β ^N^/β ^N^
33^a^	F	28	5.82	120	65.8	20.6	2.3	HKαα/--^SEA^	β ^N^/β ^N^

codons 41/42, HBB:c.126_129delCTTT; Hb, hemoglobin; IVS-II-654, HBB: c.316-197C4T; MCH, mean corpuscular hemoglobin; MCV, mean corpuscular volume; N, normal; PCR, polymerase chain reaction; RBC, red blood cell count; RDB, reverse dot blot hybridization; —, no data available.

^a^ *The hematology data and hemoglobin analysis were tested during pregnancy.*

### Using SMRT for Detection of the HKαα Allele

**FIGURE 2.**
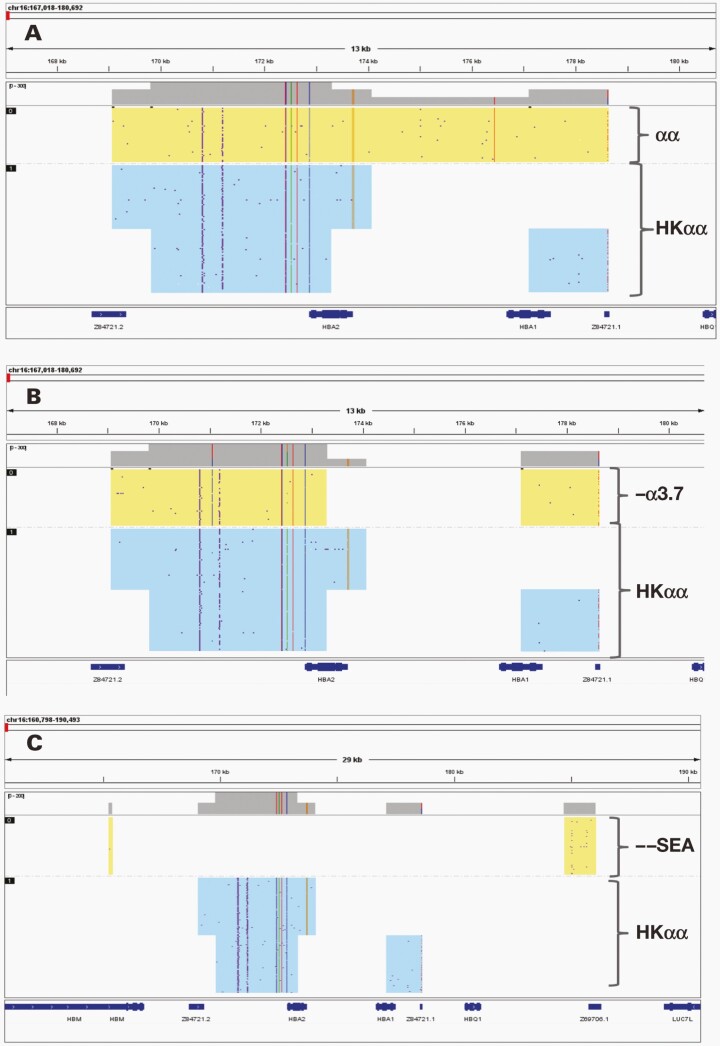
Molecular plots for the identification of α-thalassemia allele using single-molecule real-time technology. Graphs presented of samples with genotypes. A, HKαα/αα. B, –α ^37^/HKαα. C, --^SEA^/HKαα alleles.

**FIGURE 3.**
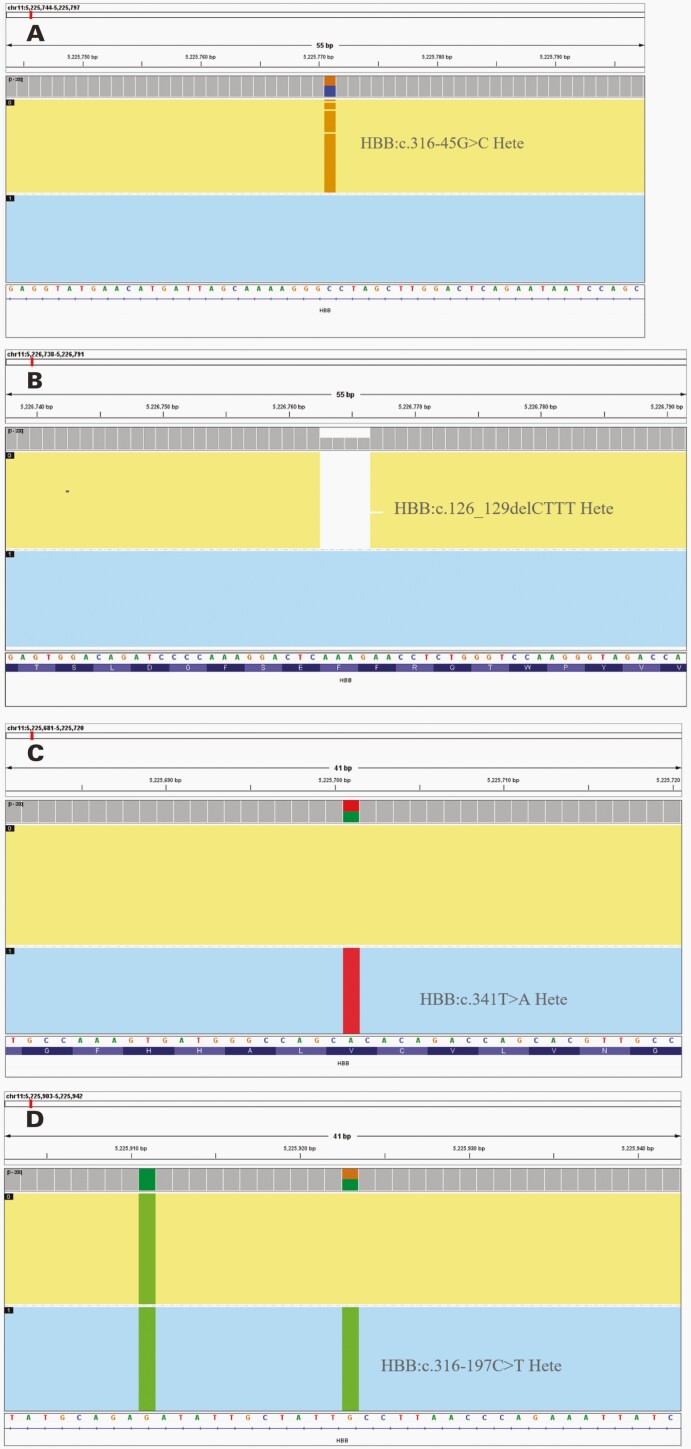
Molecular plots for the identification of β-thalassemia allele using single-molecule real-time technology. Graphs presented are of samples with genotypes. A, HBB:c.316-45G > C Hete. B, HBB:c.126_129delCTTT Hete. C, HBB:c.341T > A Hete. D, HBB:c.316-197C > T Hete.

Among the 33 cases of HKαα allele identified using SMRT, 24 cases were HKαα/αα compound normal β-thalassemia genotypes, 1 case was HKαα/αα compound HBB:c.316-197C > T Hete, 3 cases were HKαα/αα compound HBB:c.126_129delCTTT Hete, 2 cases were –α ^3.7^/HKαα compound normal β-thalassemia genotypes, 1 case was -- ^SEA^/HKαα compound normal β-thalassemia genotypes, and 1 case was -- ^SEA^/HKαα compound HBB:c.316-45G > C Hete. All of the cases of HKαα allele identified by SMRT were consistent with the conventional techniques. Further, the positive detection rate of SMRT was 100%. Notably, 2 of the detections made using SMRT differed from the conventional techniques. These were HKαα/αα compound HBB:c.341T > A Hete and -- ^SEA^/HKαα compound HBB:c.316-45G > C Hete, whereas the results of conventional techniques were HKαα/αα combined normal β-thalassemia genotypes and -- ^SEA^/HKαα compound normal β-thalassemia genotypes (**TABLE 2**, **FIGURES 2 and 3**).

**TABLE 2.** The HKαα Allele Identified by SMRT and Conventional Genetic Diagnosis Techniques

Genotype Identified by SMRT		Genotype Identified by Conventional Techniques		
α-Thalassemia Genotypes	β-Thalassemia Genotypes	α-Thalassemia Genotypes	β-Thalassemia Genotypes	No. of Samples
HKαα/αα	β ^N^/β ^N^	HKαα/αα	β ^N^/β ^N^	24
HKαα/αα	HBB:c.316-197C > T Hete	HKαα/αα	HBB:c.316-197C > T Hete	1
HKαα/αα	HBB:c.126_129delCTTT Hete	HKαα/αα	HBB:c.126_129delCTTT Hete	3
HKαα/αα	|HBB:c.341T > A Hete	HKαα/αα	β ^N^/β ^N^	1
–α ^3.7^/HKαα	β ^N^/β ^N^	–α ^3.7^/HKαα	β ^N^/β ^N^	2
--^SEA^/HKαα	β ^N^/β ^N^	--^SEA^/HKαα	β ^N^/β ^N^	1
--^SEA^/HKαα	HBB:c.316-45G > C Hete	--^SEA^/HKαα	β ^N^/β ^N^	1

SMRT, single-molecule real-time technology.

## Discussion

The HKαα allele arises due to a rearrangement in the α-globin gene cluster and contains both the –α ^3.7^ and ααα ^anti4.2^ crossover.^4^ Some of the samples diagnosed as –α ^3.7^/αα by the conventional techniques, like gap-PCR, were actually HKαα carriers, which needs to be further confirmed by 2-round nested PCR or other techniques.^5,7,8,10,14,15^ Conventional genetic diagnosis techniques can only detect target loci; they cannot detect rare and new loci directly, and the methods can be time-consuming.

Currently, there is no gold standard for detecting HKαα allele genotype(s). The most common molecular diagnostic method used is the 2-round nested PCR.^4–7^ Two-round nested PCR is an economical and effective method to detect HKαα alleles; however, it cannot identify other genotypes, and there is a high probability of cross-contamination during the second PCR amplification. Other technologies for detecting HKαα alleles include quantitative PCR, real-time PCR-based multicolor melting curve analysis, and multiplex ligation-dependent probe amplification (MLPA). MLPA is used to determine the copy number variation of genes, but it cannot determine whether the deletions and duplications are located on the same chromosome. Further, the results of MLPA are incorrect when balanced translocations of chromosomes coexist.^8,16^ Therefore, when HKαα positive results are suspected by these techniques, verification by another technique, such as Sanger sequencing, is needed to ensure accuracy.

SMRT technology can be used to test point mutations, structural variations, and triplet and fusion gene mutations without extraction or interrupting the DNA. The technique directly reads full-length gene sequences and conducts a comprehensive and accurate genetic testing for thalassemia, avoiding misjudgments caused by conventional genetic methods for diagnoses.^17–19^ For rare types of thalassemia that cannot be detected using conventional genetic technologies, SMRT can improve the diagnostic rate, which is highly valued in clinical applications.^11–13^

In this study, all cases of the HKαα allele identified by SMRT were consistent with the conventional techniques. Among the 33 HKαα allele carriers, 9 cases had normal hematological data, which were likely to be neglected in the clinic. Importantly, 2 abnormalities were detected in β-thalassemia (HBB: C.341T > A Hete and HBB: C.316-45G > C Hete) that were missed by the conventional techniques. HBB: C.341T > A Hete and HBB: C.316-45G > C Hete are considered rare β-hemoglobin variants. The c.341T > A heterozygote is a silent carrier, with c.341T > A Hete having a normal hematological phenotype and a normal reference range of HbA2 and HbF.^12,20–22^ Zhong et al^23^ hypothesized the rare variant c.316-45G > C Hete to be more likely a polymorphism. An in silico analysis verified it as a benign sequence variant without thalassemic effect. The phenotype of c.316-45G > C Hete combined with other types of α+ thalassemia may be a case of silent or mild α-thalassemia.^13^

## Conclusion

The SMRT technique showing a high detection accuracy and reproducibility that can detect not only common thalassemia variants but also variants of rare and new loci. It therefore has an important potential clinical application for thalassemia carrier screening in clinic.

## Abbreviations

SMRT: single-molecule real-time technology
HKαα: the Hong Kongαα
gap-PCR: gap-polymerase chain reaction
CCS: circular consensus sequencing
SNV: single nucleotide variant
indels: insertions and deletions
RDB: reverse dot blot hybridization
MLPA: multiplex ligation-dependent probe amplification
